# A Simple and Sensitive Method to Quantify Biodegradable Nanoparticle Biodistribution using Europium Chelates

**DOI:** 10.1038/srep13177

**Published:** 2015-09-08

**Authors:** Lindsey Crawford, Jaclyn Higgins, David Putnam

**Affiliations:** 1 School of Chemical and Biomolecular Engineering, Cornell University, Ithaca NY; 2 Department of Biological and Environmental Engineering, Cornell University, Ithaca NY; 3 Department of Biomedical Engineering, Cornell University, Ithaca NY

## Abstract

The biodistribution of biodegradable nanoparticles can be difficult to quantify. We report a method using time resolved fluorescence (TRF) from a lanthanide chelate to minimize background autofluorescence and maximize the signal to noise ratio to detect biodegradable nanoparticle distribution in mice. Specifically, antenna chelates containing europium were entrapped within nanoparticles composed of polylactic acid-polyethylene glycol diblock copolymers. Tissue accumulation of nanoparticles following intravenous injection was quantified in mice. The TRF of the nanoparticles was found to diminish as a second order function in the presence of serum and tissue compositions interfered with the europium signal. Both phenomena were corrected by linearization of the signal function and calculation of tissue-specific interference, respectively. Overall, the method is simple and robust with a detection limit five times greater than standard fluorescent probes.

Biodegradable nanoparticles (NPs), such as those derived from diblock copolymers of polylactic acid and polyethylene glycol (PLA-PEG), are valuable in the field of targeted drug delivery. The hydrophobic PLA core can entrap hydrophobic compounds to serve as a carrier for medicines that can be difficult to deliver intravenously^1^,^2^.

An important step in the development of PLA-PEG NP drug delivery systems is their detection following injection in the blood stream. Common methods to detect the biodistribution of NPs include radiolabels and fluorescent markers. Fluorescence is a safer alternative to radiolabeling; however, the autofluorescence of biological tissues makes it challenging^3^,^4^. Specifically, amino acids in biological tissues contribute to autofluorescent signals by absorbing and emitting light at frequencies that overlap with those of common fluorescent markers^5^. Therefore, the level of detection and the signal to noise ratios of common fluorescent markers is compromised leading to decreased sensitivity^6^,^7^,^8^. Another approach to the detection of NPs in tissues is to use markers that absorb and emit light in the longer wavelength near-infrared spectrum to minimize autofluorescence; however, these compounds are prone to degradation and photobleaching^9^.

As an alternative strategy to overcome autofluorescence in the detection of structures in biological tissues, time resolved fluorescence (TRF) has been explored. Elements in the lanthanide III series exhibit TRF. Their large Stokes shifts and long decay lifetimes allow them to be detected distinct from competing autofluorescence signals^10^. They are also less susceptible to photobleaching than traditional organic dyes^11^,^12^,^13^. Earlier reports have described the utility of lanthanide chelates for the detection of non-degradable NPs both *in vivo* and *ex vivo*, but degradable NPs, like those based on PLA-PEG, have unreported characteristics that makes their detection unique and should be considered to accurately quantify their concentration in tissues^14^,^15^,^16^.

This communication outlines a simple approach to the detection and quantitation of PLA-PEG NPs in tissues following intravenous injection. A hydrophobic europium chelate was encapsulated within the PLA-PEG core. The TRF of the europium-doped NPs is significantly above tissue autofluorescence, and the particles are detectable in harvested organs *ex vivo* with minimal post-processing of the tissues.

Two important characteristics of the encapsulated europium were identified when quantifying NPs in tissues. First, the TRF of europium diminishes as a second-order function upon exposure to water. Europium chelate located toward the outer surface of the particle is susceptible to water penetration along the same time scale as the biodistribution studies; therefore, the rate of signal decrease upon water exposure is an important parameter in the biodistribution calculations. Second, the TRF of europium is compromised by iron ions; therefore, the signal must be corrected for each tissue using doped tissue blanks. The described method was used to assess the biodistribution of the widely used PLA-PEG-based NP drug delivery system.

## Results

### Nanoparticle fabrication and characterization

**Figure 1:**
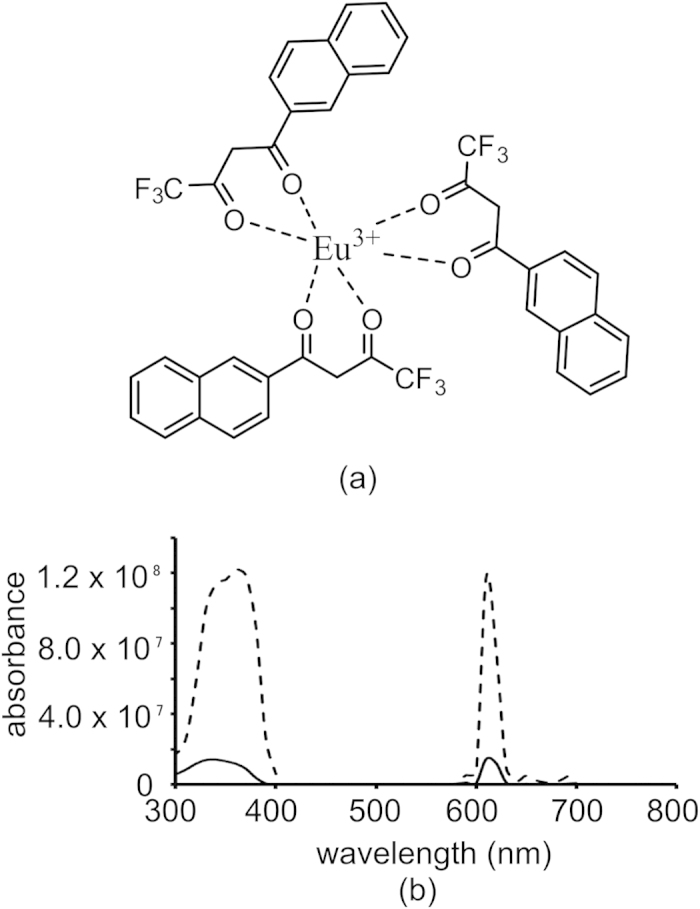
Characteristics of PLA-PEG nanoparticles doped with europium chelate. (**a**) representative structure of Eu(NTA)_3_ chelate. (**b**) excitation and emission spectra for 1 mg/mL nanoparticle suspension in PBS (solid lines) and 0.1 mg/mL chelate solution in acetone (dotted lines) under time resolved conditions.

Polylactic acid–mono methoxy polyethylene glycol (PLA-PEG) diblock copolymers were synthesized by ring opening polymerization. M_n_ was consistent between both GPC and ^1^H NMR (21,000 and 20,600, respectively). Nanoparticle fabrication yielded particles 106 ± 6.5 nm in diameter and −1.45 ± 0.25 mV surface charge with a polydispersity of 0.079 ± 0.03. Excitation and emission spectra for the particles and chelate (Fig. 1), show maximum excitation at 340 nm and a maximum emission at 610 nm. Both the unencapsulated chelate and the chelate-containing nanoparticles show a large Stokes shift, which is a hallmark characteristic of europium chelates. Release studies of europium chelate from PLA-PEG nanoparticles (Figure S1) show no detectable chelate release over nine hours.

### Sensitivity Analysis

**Figure 2:**
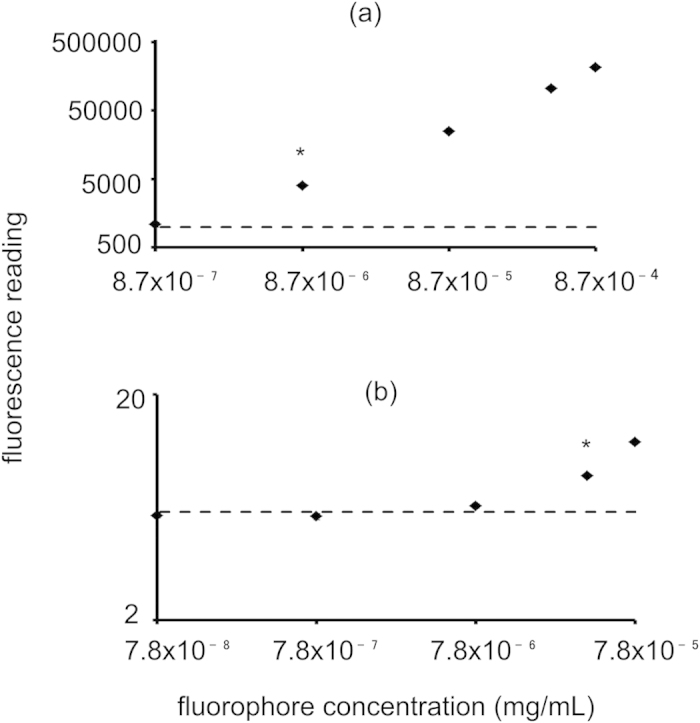
Sensitivity analysis of (**a**) Eu(NTA)_3_ doped nanoparticles and (**b**) rhodamine B doped nanoparticles. Dotted lines correspond to the sensitivity limit. Eu(NTA)_3_ doped nanoparticles are more sensitive than rhodamine B doped nanoparticles. *Lowest concentration that is statistically different from sensitivity limit at p < 0.01.

The sensitivity limit of the Eu(NTA)_3_ doped PLA-PEG NPs was determined and compared to RhoB loaded PLA-PEG NPs (Fig. 2). Encapsulation efficiencies were determined to be 87.0 ± 14% and 7.82 ± 1.7% for europium chelate and RhoB doped nanoparticles, respectively. Using the encapsulation efficiency, the europium doped NPs were detectable at as little as 8.7 × 10^−6^ mg/mL of europium chelate above the sensitivity limit compared to 3.9 × 10^−5^ mg/mL of RhoB for RhoB doped particles. This is an almost 5× increase in sensitivity using europium chelate doped nanoparticles over RhoB.

### Europium signal degradation kinetics

**Figure 3:**
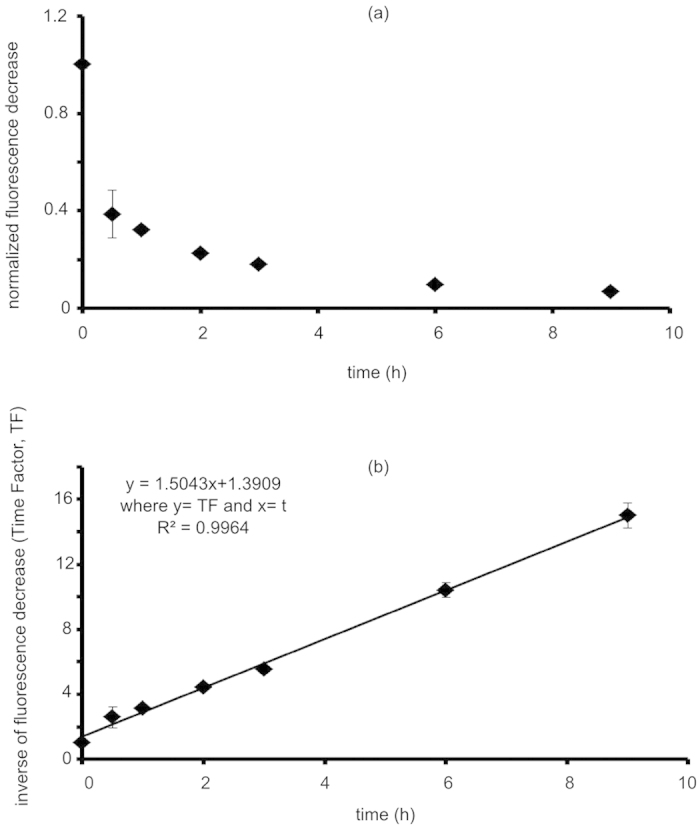
Fluorescent decrease of Eu(NTA)_3_ doped nanoparticles in plasma vs. time. (**a**) second order decrease in fluorescence. (**b**) manipulated inverse of second order decrease. Particles lose fluorescence due to the introduction of water into the chelate.

The TRF signal of europium chelates is diminished on exposure to water^17^. To account for signal changes in the context of quantifying biodistribution, the TRF of europium-doped NPs was measured in plasma (Fig. 3). The signal decay was transformed into a linear form that signified a second-order process. A straight line fit provided an equation to assess signal degradation at any given time.

### The influence of tissue on europium TRF signal

The signal of europium chelate-doped PLA-PEG particles in tissue homogenates was far above background time-resolved autofluorescence (Table 1). However, when nanoparticles were titrated into tissue homogenates, signal interference was detected and attributed to the presence of iron, although other unknown influences could not be ruled out^18^. Therefore, to account for the influence of tissue composition on the TRF signal, an interference factor was created for each tissue and used in the calculation of the final biodistribution data (Table 1). The greatest interference is seen in the liver and kidneys with a factor of 0.11 ± 0.008 and 0.11 ± 0.005, respectively. The lowest interference is in the brain with a factor of 0.37 ± 0.02.

### Nanoparticle biodistribution

**Figure 4:**
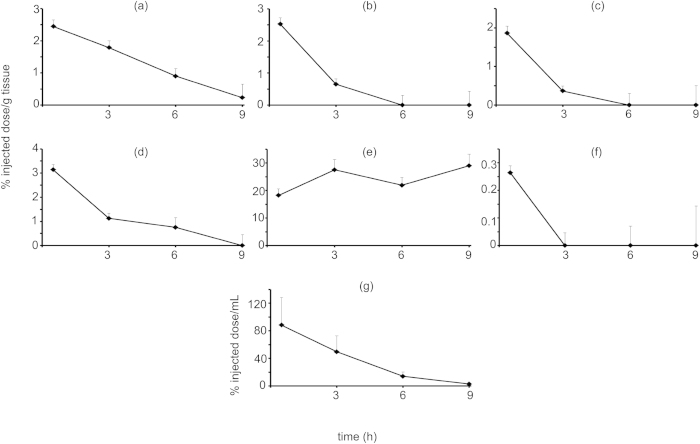
Biodistribution of Eu(NTA)_3_ doped PLA-PEG nanoparticles in (**a**) liver, (**b**) kidney, (**c**) heart, (**d**) lung, (**e**) spleen, (**f**) brain, and (**g**) blood.

Using the second order degradation TFR curve and tissue interference factor to calculate the percent of injected NP in each tissue, biodistribution curves were produced for each tissue type. Particles were detectable above background in all tissues (Fig. 4). From the area under the curves (Table 2), the highest tissue accumulation for these PLA-PEG nanoparticles was in the spleen, followed by the liver and kidney. Particles were also present in the heart, lung and brain.

## Discussion

PLA is a biodegradable polyester with degradation kinetics that can be engineered to control the rate of encapsulated drug release^19^,^20^. PEG is often used in concert with PLA NPs to enhance NP circulation time by altering the NP’s interaction with macrophages and blood proteins, acting as an inert shield against the biological milieu^21^,^22^.

PLA-PEG NPs have begun to enter clinical trials in the United States for their ability to alter the biodistribution of low molecular weight drugs and to help facilitate their transport to target therapeutic sites and reduce their side effect profiles^20^. For example, Genexol-PM is a passively targeted PEG-PLA micelle formulation of paclitaxel in Phase II clinical trials for the treatment of metastatic breast cancer. It has demonstrated an extremely promising response rate of close to 60% and is progressing through further trials^23^. PLA-PEG NPs have also shown promise as an actively targeted drug delivery system via conjugation with various targeting ligands^20^. For example, Hrkach *et al*. reported pre-clinical development of a prostate-specific membrane antigen-targeted PLA-PEG docetaxel NP for the treatment of prostate cancer which has demonstrated high success rates^24^. Additional studies show NP-specific receptor binding mediated through targeting ligands and monoclonal antibodies with several other types of cancer including ovarian, lymphoma^25^, and glioma cells^2^. There is also ongoing work with PLA-PEG NPs for drug transport across the blood brain barrier^26^ and for the treatment of spinal cord injuries^27^ – both areas where it is particular difficult to achieve high concentrations of drug.

This report outlines an easy and sensitive method to quantify biodegradable NPs following intravenous administration. The goal was to define a method to eliminate the challenges caused by signal overlap with tissue autofluorescence. The NPs reported in this study are consistent in shape and size with previous studies using the same NP fabrication methods^7^,^28^,^29^. Europium chelate encapsulation was confirmed with excitation and emission maxima appropriate for the chelate used under TRF^30^. Release experiments show no detectable europium chelate being released from the NPs implying that all measurements *in vivo* are representative of intact particles and not free chelate. Sensitivity analysis compared europium chelate doped nanoparticles to RhoB doped nanoparticles. RhoB was chosen as a model dye for comparison as it is just outside the fluorescent range that exhibits the highest autofluorescence and has been used in literature^8^,^31^,^32^. The encapsulation efficiency was low for RhoB since it is more hydrophilic than the europium chelate; however, when sensitivity is compared to fluorophore concentration, europium chelate nanoparticles were almost 5 times more detectable than RhoB nanoparticles.

Further *in vitro* testing showed a loss in NP fluorescence that followed a second-order process in the presence of plasma, likely from water penetration into the NP resulting in fluorescence quenching^17^. Studies with similar europium chelates have tried to prevent water-induced TRF quenching; however, those chelates contained an extra molecule (trioctylphosphine oxide) which occupied the remaining coordination sites of europium in hopes of preventing water quenching. Attempts in this work were made to formulate PLA-PEG NPs doped with this identical chelate, however significant aggregation occurred when this chelate was introduced^16^,^33^,^34^. Further protection from europium chelate quenching can be provided by encapsulation into a non-biodgradeable nanoparticle, such as polystyrene. Polystyrene nanoparticles encapsulating europium chelates have proven utility *in vitro*^16^,^35^, however biodegradable NPs provide advantages as drug carriers in drug delivery applications. Biodegradable nanoparticles can be naturally metabolized, leading to decreasing accumulation in the liver and spleen compared to non-biodegradable nanoparticles. This leads to less toxicity and is therefore a better option for nanoparticle drug delivery^36^. PLA-PEG, as discussed above, represents a widely studied class of biodegradable polymeric nanoparticles and thus, detection methods such as the one presented in this work are invaluable. This study aimed to use TRF to assess the biodistribution of unadulterated PLA-PEG NPs by correcting for both water quenching and tissue interference.

Detection of the particles was well above background autofluorescence in all tissues, largely due to europium’s large Stokes shift and time resolved characteristics. One challenge this method of detection does face, in context to NP biodistribution, is the interference of iron ions in various tissues. Iron has been previously reported to quench europium chelate fluorescence^18^. Therefore, to correct for the diverse concentration of iron in individual tissues, tissue-specific interference factors were obtained by doping NPs with individual organ tissue homogenates and used to accurately calculate NP biodistribution *in vivo*. To address iron variation among individuals, analysis was performed using tissues from 3 different mice. However, variation among species was not explored and should be considered in future applications.

The biodistribution studies showed that this TRF approach is able to detect PLA-PEG NPs in all harvested tissues including the liver, kidney, heart, lung, spleen, brain and blood. The data correlate well with other PLA-PEG NP biodistribution reports^37^,^38^. There is high uptake in the spleen signaling uptake by the RES.

While the reported method of TRF detection of NPs using a hydrophobic europium chelate requires correction for both water and iron interactions, the method is sensitive, simple and robust. Alternative detection methods can require laborious post processing steps (e.g., HPLC) or specialized handling of material and tissue (e.g. radiolabel), where this TRF approach requires only the use of a fluorescent plate reader. The utility of a simple detection method for PLA-PEG NPs extends beyond this initial work to future use in investigation of targeting moieties to alter the biodistribution of NPs.

## Materials

Monomethoxy polyethylene glycol (PEG, M_n_ 5000), anhydrous toluene, stannous octoate, europium chloride hexahydrate, 4,4,4-trifluoro-1-(2-naphthyl-1,3-butanedione) (NTA), acetone, dichloromethane (DCM), magnesium sulfate, poloxamer 188 solution and RIPA buffer were purchased from Sigma Aldrich (Saint Louis, MO). D,L-Lactide was purchased from TCI America (Portland, OR). Phosphate buffered saline (PBS) was purchased from Corning (Corning, NY). Ammonium hydroxide was purchased from Alpha Aesar (Ward Hill, MA). Diethyl ether was purchased from Fisher Chemicals (Waltham, MA). Methanol was purchased from Macron Fine Chemicals (Avantor Performance Materials, Center Valley, PA). Horse plasma was graciously donated by the Cornell College of Veterinary Medicine.

## Methods

### Ethics statement

All experiments were carried out in accordance with safety and animal welfare guidelines and protocols approved by Cornell University. All experimental animal protocols were approved by the IACUC of Cornell University (IACUC protocol #2012-0034).

### PEG azeotropic distillation

Monomethoxy PEG (50 g, M_n_ 5000) was dissolved in 200 mL of anhydrous toluene with slight heating. Distillation was carried out under reflux at 128 °C for 2 hours using a Dean Stark trap. The remaining toluene was removed by rotoevaporation and the PEG product dried under high vacuum.

### PLA-PEG diblock copolymer synthesis and characterization

Lactide was recrystallized once from methanol and dried under high vacuum prior to the reaction. PEG (400 mg, 0.08 mmol) and lactide (1843 mg, 12.8 mmol) were dissolved in anhydrous toluene (6 mL) under argon in a 25 mL Schlenk flask. The reaction vessel was placed in a 111 °C mineral oil bath. Upon the initiation of toluene reflux (111 °C), toluene (2 mL) containing stannous octoate (0.14 mmol) was added all at once with stirring. The reaction was allowed to reflux with stirring over 24 hours, after which excess toluene was removed by rotoevaporation until an oily liquid remained and the product collected by precipitation into excess diethyl ether with stirring. The product was collected by filtration, dried overnight under high vacuum and stored at room temperature in a vacuum dessicator until characterization and nanoparticle production. The molecular weight of the diblock copolymer was determined using both GPC (polystyrene standards) and ^1^H NMR. ^1^H NMR was used to confirm PLA-PEG structure ppm: 5.20 (m, CH PLA), 3.51 (s,CH_2_CH_2_ PEG), 3.24 (s, CH_3_ PEG), 1.45 (m, CH_3_ PLA).

### Europium(III)tris(4,4,4-trifluoro-1-(2-naphthyl-1,3-butanedione)) chelate synthesis

The europium(III)tris(4,4,4-trifluoro-1-(2-naphthyl-1,3-butanedione)) (Eu(NTA)_3_) chelate was synthesized as previously reported^35^. In brief, NTA (800 mg) was dissolved in 75 mL of ethanol (75 mL) and ammonium hydroxide (20.4 mL of 28%) with stirring. On complete dissolution, a solution of 366 mg of europium chloride hexahydrate (366 mg) in DI water (10 mL) was added dropwise to the NTA solution. The chelate was allowed to form overnight by ethanol evaporation with stirring at room temperature. The resulting solution was then extracted with DCM, the organic layer was then isolated and washed with DI water three times. The DCM solution was dried over magnesium sulfate, removed by rotoevaporation, and the resulting solid was dried under high vacuum.

### Nanoparticle fabrication and characterization

PLA-PEG (200 mg) was dissolved in DCM (2 mL) then vortexed with a DCM solution of Eu(NTA)_3_ (1 mg/mL, 2 mL total). The solution was then diluted with acetone (16 mL), vortexed and added dropwise into MilliQ water (20 mL) with stirring. The organic solvents were removed by rotoevaporation and the nanoparticles collected by ultracentrifugation using a Bruker LE-80 ultracentrifuge at 20,000 rpm for 30 minutes. The nanoparticles in the pellet were washed twice with MilliQ water and resuspended in a final volume of 1 mL of MilliQ water. Poloxamer 188 (40 μL of 10% solution) was added prior to lyophilization overnight.

For nanoparticle characterization, a particle suspension (1 mg/mL) in PBS was sonicated in a VWR B1500A-MTH (Radnor, PA) bath sonicator for 30 minutes and flowed through a 0.45 μm filter before analysis. Size and polydispersity were measured with a Malvern NanoZS (United Kingdom). Excitation and emission spectra for the nanoparticles were taken on a SpectraMax GeminiEM fluorescent plate reader (Molecular Devices, Sunnyvale, CA) under time resolved conditions (start time 250 μs, end time 1450 μs) using a white 96 well plate and compared to excitation and emission spectra of 0.1 mg/mL solution of the chelate alone in acetone.

The decrease in TRF of the nanoparticles was determined in horse plasma. A nanoparticle stock suspension (1 mg/mL in PBS) was sonicated for 30 min. (to parallel the biodistribution studies), flowed through a 0.45 μm filter, then diluted to 0.1 mg/mL with fresh horse plasma that was obtained from heparinized horse blood. The particles in plasma were incubated at 37 °C and TRF was measured at 0.5, 1, 2, 3, 6 and 9 hours in triplicate. The data was transformed into a plot of inverse fluorescence decrease vs. time which was fit with a straight line. The straight line fit was then used to calculate the decrease in fluorescent signal for any time.

Release of europium chelate from the particles was also determined. A 1 mg/mL solution of nanoparticles in PBS was sonicated for 30 minutes. 10,000 MWCO Slide-A-Lyzer^TM^ devices were soaked in 1.25 mL PBS in 1.75 mL Eppendorf tubes. Once the nanoparticles were sonicated, they were filtered through a 0.45 μm membrane and 200 μL of particle suspension was added to each dialysis chamber. The capped chambers were shaken at 37 °C and at 0, 0.5, 3, 6 and 9 hours the time resolved fluorescence was measured in the bottom solution to determine amount of chelate released. Each time point was performed in triplicate.

### Nanoparticle sensitivity

The sensitivity of detection above tissue autofluorescence of the europium chelate doped NPs were tested against rhodamine B doped NPs produced by the same procedure. A solution (1 mg/mL) of NPs was suspended in PBS and sonicated for 30 minutes, flowed through a 0.45 μm filter and diluted to 0.1, 0.05, 0.01, 0.001, 0.0001 mg/mL with liver homogenate. Liver homogenate was used as a representative tissue since it has the highest autofluorescence and greatest interference. The europium chelate doped NP dilutions were read under the time resolved conditions mentioned previously. Rhodamine B doped NP dilutions were read at excitation and emission wavelengths of 540 and 625 nm, respectively. The fluorescent values were plotted on a logarithmic scale along with a dotted line representing sensitivity of detection. Sensitivity of detection was defined as the average value of autofluorescence plus 3 standard deviations. Experiments were performed in triplicate. To compare the sensitivity as it relates to encapsulated fluorophore, encapsulation efficiency was calculated for both europium chelate doped NPs and RhoB doped NPs. The fluorescence of a 1 mg/mL solution of fluorophore containing NPs in acetone was compared to the fluorescence of a 0.01 mg/mL solution of free fluorophore in acetone. The encapsulation efficiency was then used to calculated the corresponding fluorophore concentration used in the sensitivity analysis. Statistical significance was determined using a Student’s t-test at a significance level of p < 0.01.

### *In vivo* biodistribution

Seven week old ND4 Swiss Webster mice were purchased from Harlan Sprague Dawley. A suspension of nanoparticles (1 mg/mL) in sterile PBS was sonicated for 30 minutes and then flowed through a 0.45 μm filter. A 200 μL aliquot of the nanoparticle suspension was injected intravenously through the tail vein. At 0.5, 3, 6 and 9 hours, five mice were euthanized by CO_2_ inhalation under an approved IACUC protocol (Cornell University, protocol number 2012-0034). Blood was drawn via cardiac puncture and their liver, kidney, heart, lung, spleen and brain removed. Each organ was weighed, homogenized in 2 mL of RIPA buffer and the resulting mixtures were centrifuged at 800 × g for 10 minutes. Time-resolved fluorescence was measured in three separate samples of the homogenate supernatant for each of the five mice per organ. Percent injected dose was determined by subtracting previously recorded blank readings for each organ type. This value was then divided by the TRF of injected nanoparticles along with the interference factor. The interference factor was determined by doping 20 μL of nanoparticle suspension in PBS into 180 μL of various untreated tissue homogenates. The solutions were read under time resolved conditions and the tissue value was adjusted according to Eq. 2. The sensitivity limit (autofluorescent value +3 standard deviations) was subtracted from the raw data. This value was then divided by the control of nanoparticles in PBS alone and the interference factor. The final adjustment was made by multiplying the time factor obtained from the second order equation. The variables in the equation are: RD = raw data, SL = sensitivity limit, ID = injected dose, IF = interference factor, g tissue = gram of tissue and TF = time factor. Time factor was calculated by plugging in the appropriate time value into the straight line fit of the inverse fluorescent decrease vs. time graph (Eq. 1). Area under the curve from 30 minutes to 9 hours was calculated by the trapezoid rule.


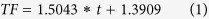



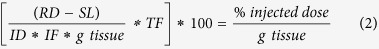


## Additional Information

**How to cite this article**: Crawford, L. *et al.* A Simple and Sensitive Method to Quantify Biodegradable Nanoparticle Biodistribution using Europium Chelates. *Sci. Rep.* **5**, 13177; doi: 10.1038/srep13177 (2015).

## Supplementary Material

Supplementary Information

## Figures and Tables

**Table 1:** Time resolved fluorescence signal and associated tissue interference (arbitrary units).

Organ	AutoFluorescentReading	Doped FluorescentReading	InterferenceRatio
NP	—	2.77 × 10^6^ ± 3.2 × 10^4^	1.0 ± 0.02
Liver	587 ± 43	3.07 × 10^5^ ± 2.3 × 10^4^	0.11 ± 0.008
Kidney	514 ± 51	2.98 × 10^5^ ± 1.4 × 10^4^	0.11 ± 0.005
Heart	476 ± 40	7.64 × 10^5^ ± 7.8 × 10^5^	0.28 ± 0.03
Lung	522 ± 28	4.23 × 10^5^ ± 4.3 × 10^3^	0.15 ± 0.002
Spleen	540 ± 68	5.64 × 10^5^ ± 1.1 × 10^5^	0.20 ± 0.04
Brain	530 ± 39	1.03 × 10^6^ ± 5.9 × 10^4^	0.37 ± 0.02
Blood	505 ± 51	5.04 × 10^5^ ± 2.3 × 10^5^	0.18 ± 0.08

The interference ratio of individual tissue homogenates compared to nanoparticle (NPs) suspension was determined by dividing the time resolved fluorescence of NPs doped into various tissue homogenates with NPs in PBS.

**Table 2:** Area under the curve from 30 minutes to 9 hours calculated with the trapezoid rule.

Organ	AUC (%ID h/g)
Liver	11.0 ± 1.6
Kidney	4.96 ± 1.6
Heart	3.33 ± 1.6
Lung	9.28 ± 2.0
Spleen	208 ± 20
Brain	0.33 ± 0.4
Blood*	293 ± 103

*Blood values represented as % ID/mL.
