# Supplementary material for: A Simple and Sensitive Method to Quantify Biodegradable Nanoparticle Biodistribution using Europium Chelates
Source: Sci Rep. 2015 Sep 8;5:13177. doi: 10.1038/srep13177 (PMC4561907; doi:10.1038/srep13177)
Supplement: Supplementary Information [file srep13177-s1.pdf]

A Simple and Sensitive Method to Quantify Biodegradable Nanoparticle Biodistribution using Europium Chelates

Lindsey Crawford[1], Jaclyn Higgins[2] and David Putnam[1,3*]

[1]School of Chemical and Biomolecular Engineering, [2]Department of Biological and Environmental Engineering, [3]Department of Biomedical Engineering,

Cornell University, Ithaca NY

[*]To whom correspondence should be addressed. Department of Biomedical Engineering, 147 Weill Hall, Cornell University, Ithaca, NY 14853. dap43@cornell.edu

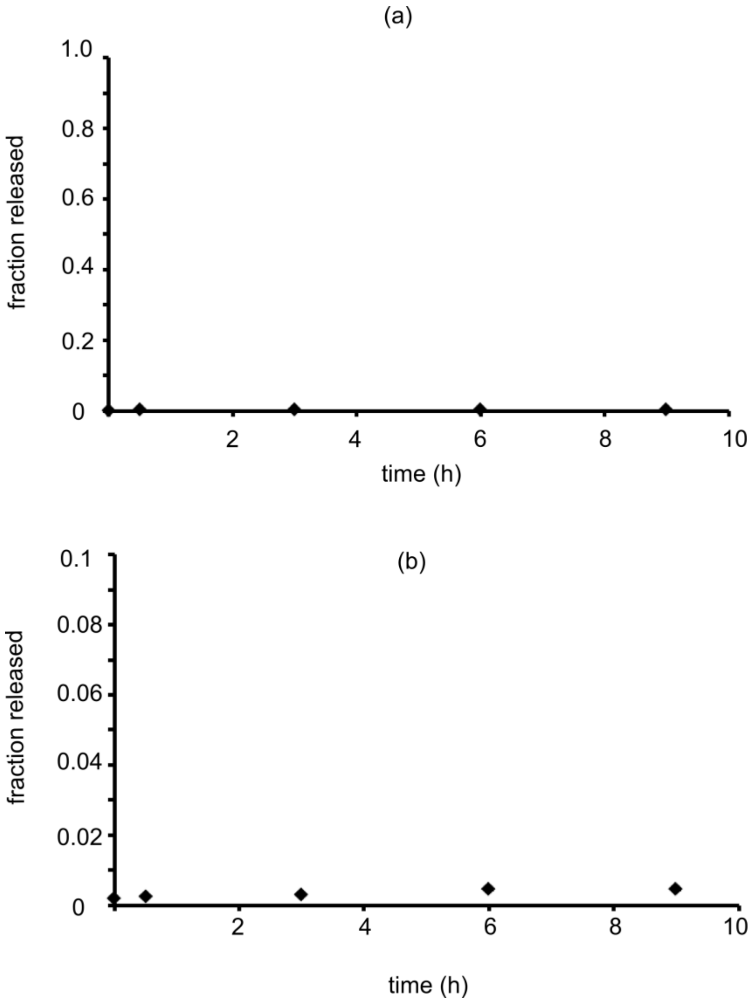

Figure S1. Release data for $Eu(NTA)_3$ doped PLA-PEG nanoparticles. (a) release data on a 100% scale. (b) release data on a 10% scale. Neither scale shows any detectable chelate released.
